# STAFF PERCEPTIONS OF INVOLUNTARY NURSING HOME CLOSURE AND RELOCATION PROCESSES

**DOI:** 10.1093/geroni/igz038.2580

**Published:** 2019-11-08

**Authors:** Raven H Weaver, Karen A Roberto, Nancy Brossoie, Pamela B Teaster

**Affiliations:** 1 Washington State University, Pullman, Washington, United States; 2 Virginia Tech, Blacksburg, Virginia, United States

## Abstract

Involuntary nursing home closures happens infrequently, but when they do occur, they impact residents, their family, and facility staff. During the transition, residents’ care needs are of primary concern, yet few studies have examined the centrality of the actions of staff to residents’ relocation adjustment. This paper examined staff perceptions of the involuntary relocation process for 132 residents after a facility lost its Medicaid certification because of low quality performance. Interviews were conducted with 34 staff (e.g., administrators, nurses, social workers) from 21 receiving facilities. Using content analysis, we identified challenges that hindered relocation and affected resident/family experiences. Receiving facility staff perceived undue distress and hardship on residents and family members because of inadequate notification about the situation. Limited, untimely, and poor communication led to residents being uninformed or unprepared for moving. The efficiency and effectiveness of the resident discharge process was also viewed as unacceptable. Minimal documentation in residents’ charts hampered the coordination of resident moves. Receiving facility staff offered recommendations for decertified facilities and receiving facilities to improve the relocation experience including the need for open communication, thoughtful and early engagement in the process, and transparent and timely interactions. Findings suggest that staff are well-positioned for active involvement in the relocation process and should facilitate deliberate and strategic planning, decision-making, and communication with residents and their relatives. Resident-centered policies are needed to improve the involuntary relocation process and give voice to remaining/receiving staff, both of whom are integral to residents’ support system.

